# Band tail state related photoluminescence and photoresponse of ZnMgO solid solution nanostructured films

**DOI:** 10.3762/bjnano.11.75

**Published:** 2020-06-12

**Authors:** Vadim Morari, Aida Pantazi, Nicolai Curmei, Vitalie Postolache, Emil V Rusu, Marius Enachescu, Ion M Tiginyanu, Veaceslav V Ursaki

**Affiliations:** 1 D.Ghitu Institute of Electronic Engineering and Nanotechnologies, Chisinau MD-2028, Republic of Moldova; 2 Center for Surface Science and NanoTechnology, University Politehnica of Bucharest, 060042-Bucharest, Romania; 3 Institute of Applied Physics, Chisinau MD-2028, Republic of Moldova; 4 National Center for Materials Study and Testing, Technical University of Moldova, Chisinau MD-2004, Republic of Moldova

**Keywords:** aerosol spray pyrolysis deposition, energy band tails, photodetector, photoluminescence, photosensitivity, spin coating, thin films, ZnMgO semiconductor alloy

## Abstract

A series of Zn_1−_*_x_*Mg*_x_*O thin films with the composition range *x* = 0.00–0.40 has been prepared by sol–gel spin coating on Si substrates with a post-deposition thermal treatment in the temperature range of 400–650 °C. The morphology of the films was investigated by scanning electron microscopy and atomic force microscopy while their light emission properties were studied by photoluminescence spectroscopy under excitation at 325 nm. It was found that annealing at 500 °C leads to the production of macroscopically homogeneous wurtzite phase films, while thermal treatment at higher or lower temperature results in the degradation of the morphology, or in the formation of ZnO particles embedded into the ZnMgO matrix, respectively. Local compositional fluctuations leading to the formation of deep band tails in the gap were deduced from photoluminescence spectra. A model for the band tail distribution in the bandgap is proposed as a function of the alloy composition. Thin films were also prepared by aerosol spray pyrolysis deposition using the same sol–gel precursors for the purpose of comparison. The prepared films were tested for photodetector applications.

## Introduction

The ZnMgO solid solution system is of interest due to the possibility to tailor many important physical properties by varying their composition. This alloy system covers a wide ultraviolet (UV) spectral range between the direct bandgaps of 3.36 eV for ZnO and 7.8 eV for MgO at room temperature, making it very attractive for short-wavelength optical applications such as UV detectors [1–5] and light emitters [6–9].

Various techniques have been used for the preparation of ZnMgO films such as radio-frequency plasma-assisted molecular beam epitaxy (RF-MBE) [2,7,10–11], DC [12–13] and RF [1,3,6] magnetron sputtering, pulsed laser deposition (PLD) [14–15], plasma-enhanced atomic layer deposition (PE-ALD) [16], chemical vapor deposition (CVD) [17], metal–organic chemical vapor deposition (MOCVD) [18–19], hydrothermal [4], chemical bath deposition (CBD) [20], sol–gel spin coating [21–29], and spray pyrolysis [28–34]. Among these techniques, the sol–gel spin coating method has the advantage of ensuring easy control and handling of chemicals and substrates, as well as excellent control over stoichiometry. Because the process does not require vacuum conditions and can be performed at low temperature, it is suitable for the fabrication of high quality, large area thin films at a fast rate and low cost. This method also offers the possibility for easy doping and preparation of homogeneous films with good electrical and optical properties.

The films are prepared on various substrates such as ZnO [6], MgO [17], Si [2–4,23], CaF_2_ [12], Al_2_O_3_ [18], sapphire [7,10–11,13–16,19,31–32], glass and quartz [1,20–21,23–30,33–34]. The choice of the substrate is determined by the application. In particular, glass, quartz or sapphire substrates are usually used for photodetectors in the metal–semiconductor–metal (MSM) configuration, including Schottky photodetectors [1,19,24–25,28–32]. A comparison of MSM photodetectors based on ZnMgO films prepared by spin coating and spray pyrolysis performed recently revealed that the sensitivity of the structures prepared by spin coating is higher as compared to those obtained by spray pyrolysis, while the photoresponse to UV irradiation is faster in devices based on spray pyrolysis films [29].

Solar-blind UV photodetectors with the highest responsivity to date were demonstrated on sapphire substrates by introducing ZnO or Al_2_O_3_ buffer layers [11,16]. With respect to photodetectors with p–n junctions, some photodetectors have been demonstrated on p-type Si [2,4] because p-type doping is still a big challenge to ZnO-based semiconductors. Liang et al. demonstrated a ZnMgO/p-Si heterojunction solar-blind UV photodetector with a BeO buffer layer [35].

In terms of the crystal structure of ZnMgO films used in photodetectors, three types of structures are used, namely, hexagonal wurtzite structure (w-ZnMgO), cubic rock salt structure (c-ZnMgO) and films with mixed-phase (m-ZnMgO) [5]. Since the crystal structure of the alloy changes from w-ZnMgO to c-ZnMgO with increasing Mg content, the coexistence of two structures in ZnMgO films is unavoidable in the structure transformation process, in a certain interval of Mg concentrations. The phase segregation process was investigated in detail by means of X-ray diffraction, element-speciﬁc near-edge X-ray absorption ﬁne structure (NEXAFS), electron dispersive spectroscopy (EDS), atomic force microscopy (AFM), UV–vis spectroscopy, photoluminescence (PL) and resistivity measurements in Zn_1−_*_x_*Mg*_x_*O thin ﬁlms deposited by the sol–gel spin-coating route in the composition range *x* = 0.00–0.40 [23]. It was found that the phase segregation manifests itself starting at a Mg content of *x* = 0.25. However, the results showed that ﬁlms are deposited with wurtzite structure as the dominant phase even after phase segregation in the investigated Mg concentration interval. The issue of phase segregation was also investigated via selective resonant Raman scattering in a wider composition range of *x* = 0.00–0.78 for Zn_1−_*_x_*Mg*_x_*O thin ﬁlms grown by reactive DC magnetron co-sputtering [12]. It was shown that this investigation technique is highly sensitive for the detection of embedded structural inhomogeneities, and it was found that the phase segregation occurs in the range of *x* = 0.35–0.65 with coexistence of both wurtzite and NaCl structures.

In addition to crystallographic structure fluctuations, compositional fluctuations at the microscopic level are even more likely in Zn_1−_*_x_*Mg*_x_*O alloys. Compositional fluctuations have been deduced from photoluminescence (PL), photoluminescence excitation (PLE) and optical absorption (OA) spectroscopy investigations in w-ZnMgO films produced by RF-MBE in the composition range *x* = 0.00–0.37 [36] and *x* = 0.27–0.55 [10], respectively. The observed Stokes shifts were indicative of the presence of band tail states introduced by alloying, while the “S-shaped” temperature dependence of the maximum PL emissions was explained in terms of exciton localization in potential traps induced by Mg compositional fluctuations. The fluctuations in the local arrangement of Mg and Zn atoms have been also recently investigated by means of cathodoluminescence (CL) and OA spectroscopy in c-ZnMgO films produced by CVD in the composition range *x* = 0.61–0.81 [17], where a relatively large Stokes-like shift of 0.7–0.8 eV was observed. The understanding of the influence of compositional fluctuations in sol–gel spin-coated ZnMgO thin films on their properties is of particular importance. Towards this goal, the present study explores the PL characteristics of Zn_1−_*_x_*Mg*_x_*O films in the composition range *x* = 0.00–0.40, under excitation with sub-bandgap photon energies from the 325 nm line of a He–Cd laser. Some possible applications of these films as UV photodetectors are also discussed.

## Results and Discussion

**Figure 1:**
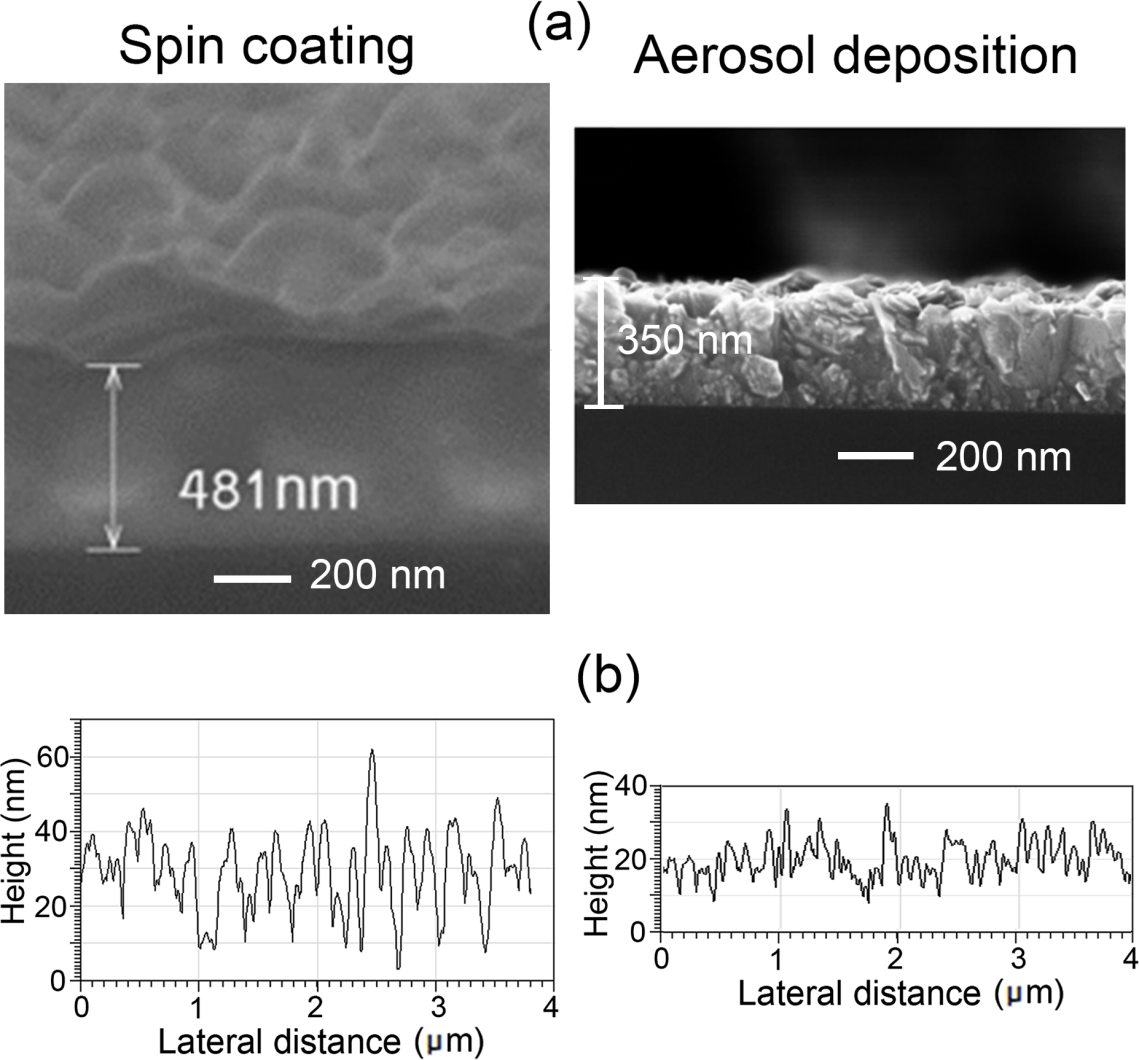
a) SEM images of ZnMgO films deposited on p-Si substrates by spin coating (left column) and aerosol deposition (right column) methods. b) Graphical representations of the AFM profiles for ZnMgO films.

Figure 1a compares the morphology of ZnMgO films deposited by spin coating and aerosol spray pyrolysis methods. Both methods produce thin films with uniform morphology. However, the roughness of films prepared by spin coating is larger as compared to those prepared by aerosol spray pyrolysis. The roughness parameters of films were determined from the analysis of AFM images as published in our previous paper [28]. Graphical representations of the AFM profiles for films prepared by spin coating and aerosol spray pyrolysis are presented in Figure 1b. The RMS values deduced from the AFM profiles were found to be of 12 nm and 5 nm for ZnMgO films prepared by spin coating and aerosol deposition, respectively.

**Figure 2:**
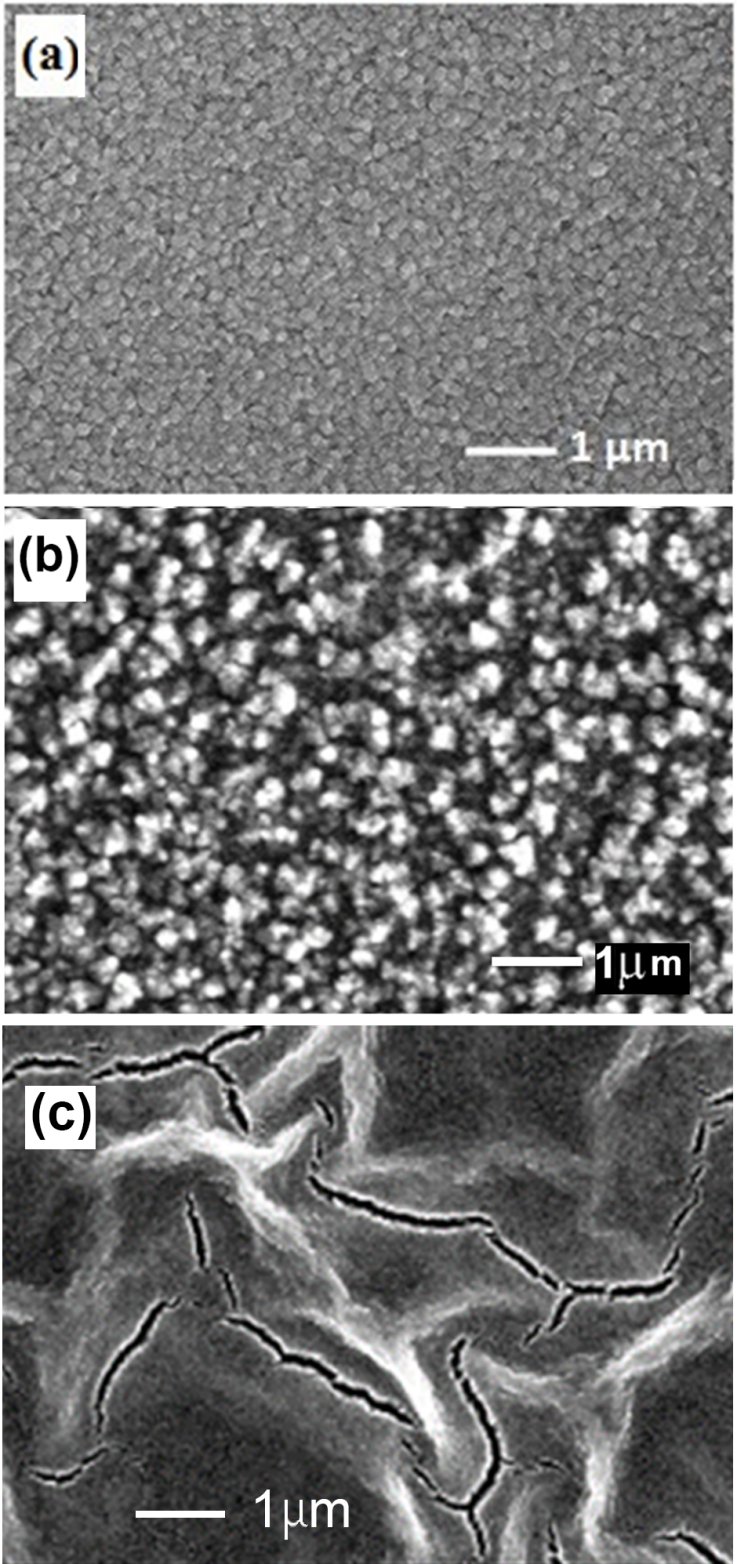
a) SEM image of a ZnMgO film prepared by aerosol spray pyrolysis. b) SEM image of a ZnMgO film prepared by spin coating and annealed at 500 °C. c) SEM image of a ZnMgO film prepared by spin coating and annealed at 650 °C.

As described in the experimental section, the thickness of films prepared by spin coating is determined by the number of cycles applied. One should note that the morphology of films deposited by spin coating and subjected to post-deposition annealing at 400 °C and 500 °C is similar. However, the morphology degrades for films annealed at temperatures higher than 600 °C. Figure 2 compares the surface morphology of films prepared by aerosol spray pyrolysis and spin coating annealed at 500 °C with the morphology of the film prepared by spin coating annealed at 650 °C. The analysis of the morphology in Figure 2a and Figure 2b corroborate the results of the AFM analysis revealing a larger roughness of films prepared by spin coating as compared to those prepared by aerosol spray pyrolysis. At the same time, the annealing of films at 650 °C (see Figure 2c) leads to deterioration of the morphology resulting in numerous cracks.

We suppose that the difference in roughness of films prepared by the two methods is determined by the specific features of the technology. Namely, the deposition of films by spray pyrolysis occurs in a single technological step, while ten cycles are applied in spin coating, and the deposited film is annealed in the eleventh step. Apart from that, the deposition of films with spray pyrolysis is performed at a relatively high temperature of the substrate (400–650 °C), while the substrate is maintained at room temperature during the spin coating.

The influence of the film thickness on the morphology was not investigated specifically in this paper. However, it was observed that the film roughness increases with increasing film thickness from 100 nm to 500 nm. Thus, the morphology parameters, as well as the electrical parameters, should be compared for films with as close as possible thicknesses. The roughness of films prepared by spin coating is also determined by the viscosity and concentration of the solution used as well as by the rotational speed of the substrate.

**Figure 3:**
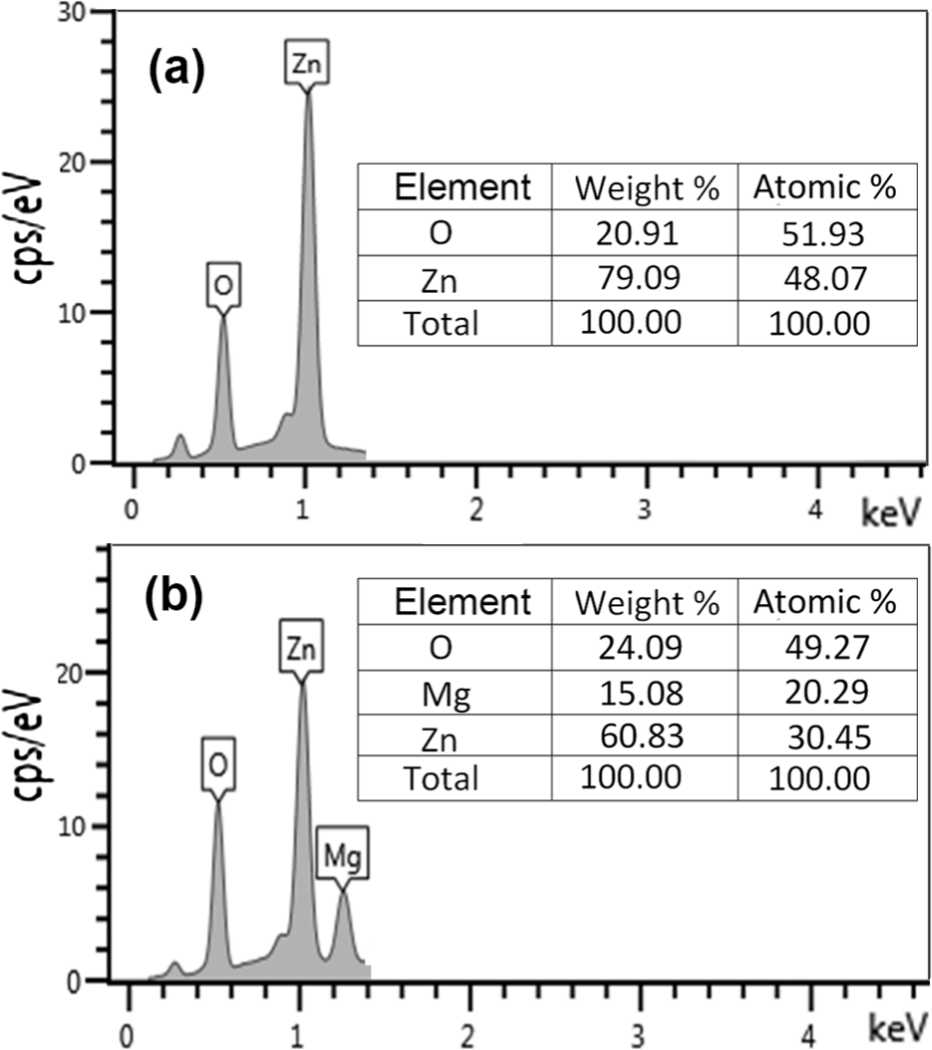
Elemental composition of a ZnO (a) and a Zn_0.6_Mg_0.4_O (b) film determined by EDAX analysis.

The composition of the prepared films was determined by energy dispersive X-ray analysis (EDAX). Examples of the elemental composition analysis are presented in Figure 3 for ZnO and Zn_0.6_Mg_0.4_O films. The results of measurements demonstrate stoichiometric compositions within limits of the errors defined by instrumental accuracy.

**Figure 4:**
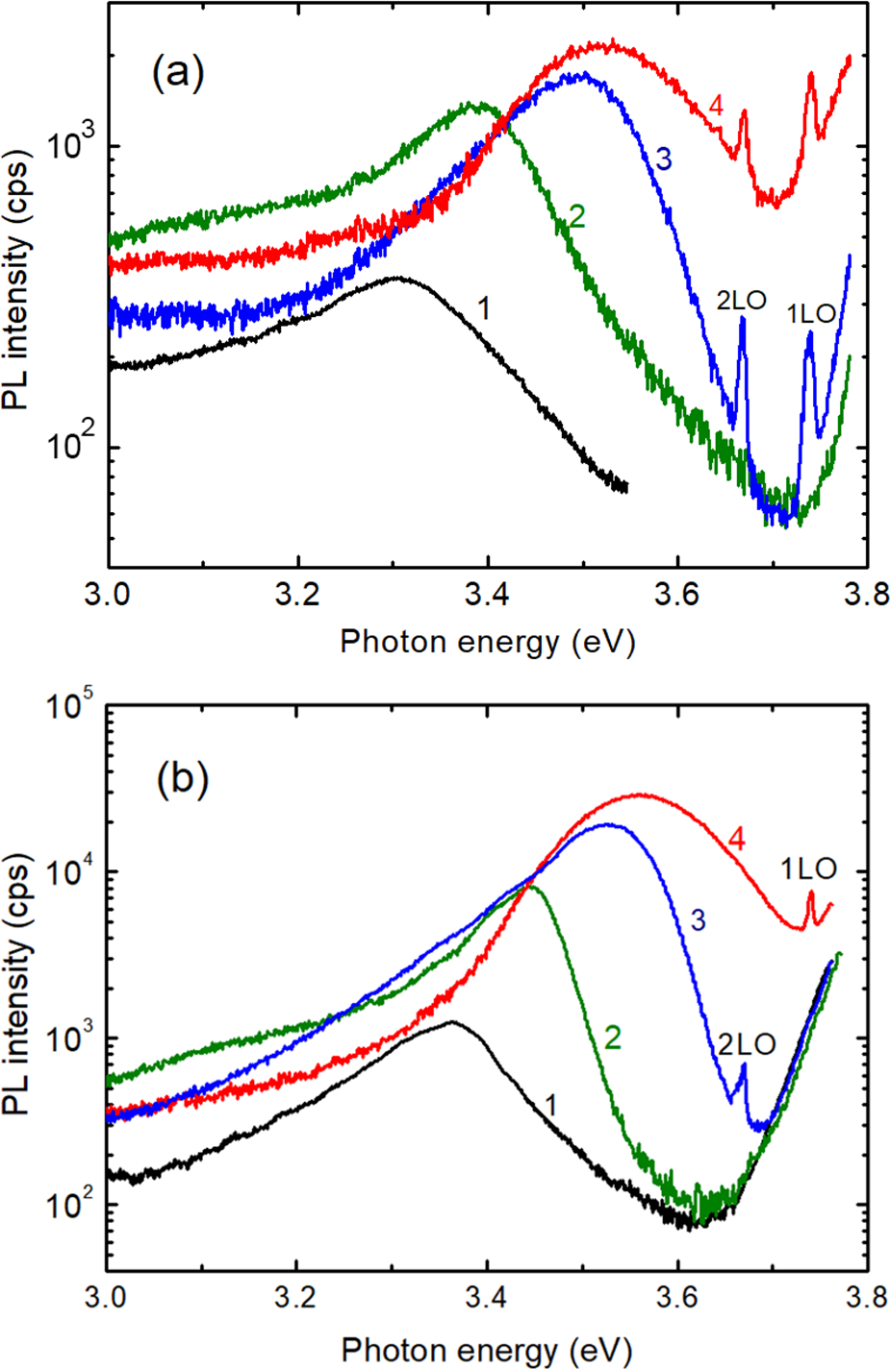
PL spectra of Zn_1−_*_x_*Mg*_x_*O films deposited by spin coating with *x* values of 0.00 (1); 0.05 (2); 0.15 (3); and 0.40 (4), annealed at 500 °C and measured at a) 300 K and b) 20 K.

The luminescence was investigated in films annealed at 400 °C and 500 °C. As seen from Figure 4, the PL spectra of films annealed at 500 °C consist of a broad emission band at both room temperature and low temperatures, which shifts to higher photon energies with increasing Mg content in the alloy. However, the position of the PL band does not follow the increase of the alloy bandgap with increasing *x* value. The higher the *x* value, the larger the difference between the bandgap and the PL band maximum.

Table 1 compares the position of the PL band with the bandgap of the alloy. Moreover, the luminescence is excited by the photon energy (3.81 eV) much lower than the bandgap for the thin film with the x value of 0.40 (4.28 eV).

**Table 1:** The PL band spectral position and the bandgap value for ZnMgO films at room temperature.

*x* value	PL band maximum (eV)	Alloy bandgap (eV)^a^

0.00	3.30	3.36
0.05	3.39	3.47
0.15	3.50	3.70
0.40	3.53	4.28

^a^From [37].

The same is true for the luminescence measured at low temperature (Table 2). The bandgap of the alloy at low temperature was recalculated from the known values at room temperature, taking into account that the bandgap increases by around 80 meV with the decrease of temperature from 300 K to 20 K [38].

**Table 2:** The PL band spectral position and the bandgap value for ZnMgO films at 20 K.

*x* value	PL band maximum (eV)	Alloy bandgap (eV)

0.00	3.36	3.44
0.05	3.45	3.55
0.15	3.53	3.78
0.40	3.56	4.36

These observations are explained by the formation of large band tails in the density of states of solid solutions. It was shown that large random local-potential fluctuations occur in highly doped and compensated semiconductors [39] and solid solutions [40] due to the microscopic inhomogeneity caused by impurity distribution in the first case and composition distribution in the second case. This spatially fluctuating band structure results in the formation of deep band tails in the gap.

**Figure 5:**
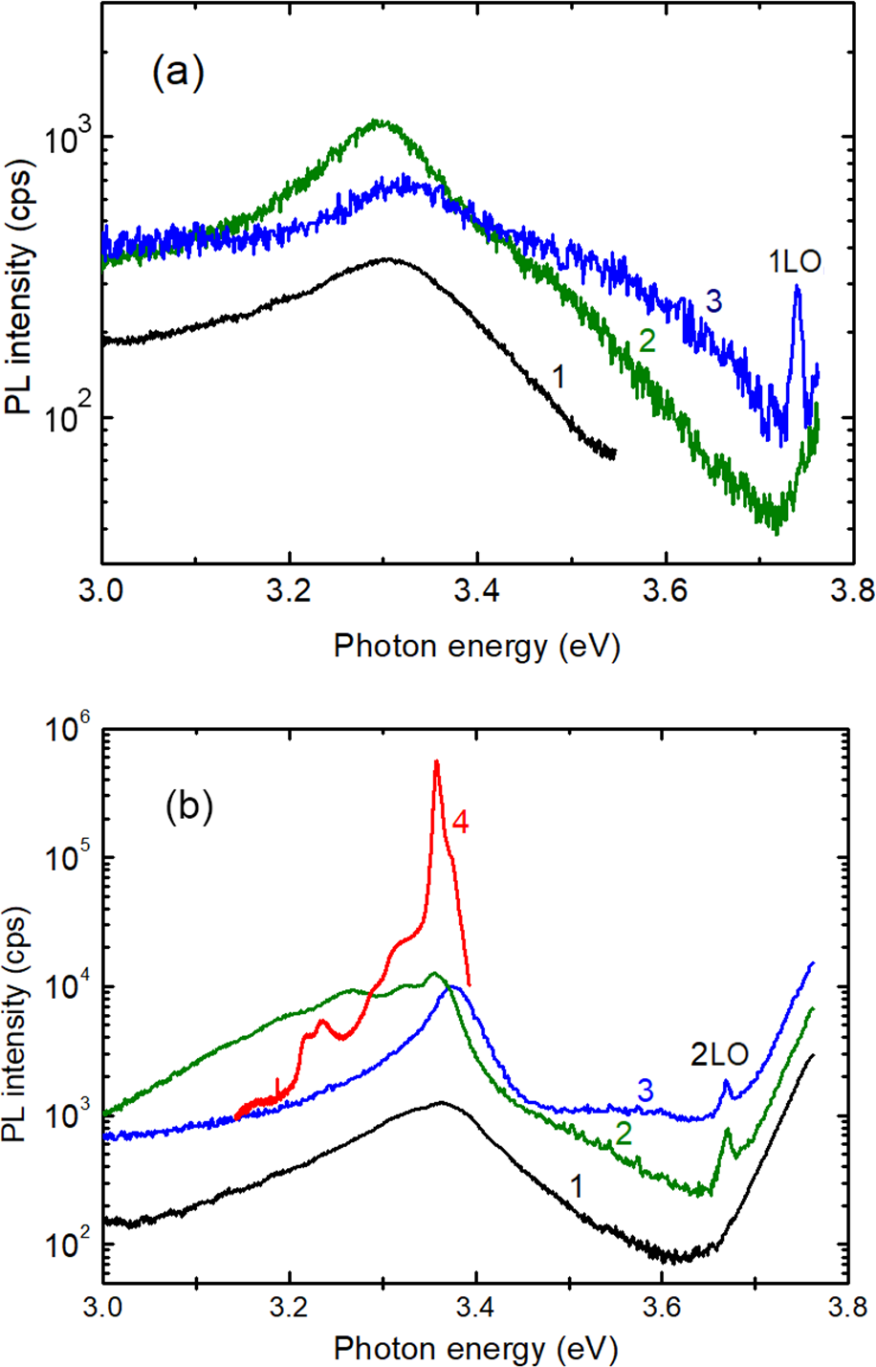
PL spectra of Zn_1−_*_x_*Mg*_x_*O films deposited by spin coating with x values of 0.00 (1); 0.10 (2); and 0.25 (3), annealed at 400 °C and measured at a) 300 K and b) 20 K. For comparison, the spectrum of a bulk ZnO single crystal is shown by curve (4).

As for the samples annealed at 400 °C, the luminescence spectra revealed the presence of two PL bands, as shown in Figure 5, which is indicative of the presence of two components in the samples. The lower energy PL band comes from ZnO crystallites embedded into the ZnMgO alloy matrix, which is responsible for the high energy broad PL band. To demonstrate that the low energy PL band is related to ZnO crystallites, it is compared to the spectrum of a high quality ZnO crystal measured at low temperature (curve 4 in Figure 5b). One can see that the spectral position of the main PL bands coincides well. In the ZnO single crystal, the main PL band at 3.359 eV is associated with the radiative recombination of neutral donor bound excitons (D^0^X) [38,41–42]. The shoulder at higher photon energies is due to the recombination of free A excitons, while the PL bands at lower photon energies represent the LO-phonon replicas of the AX and D^0^X bands at 3.29–3.31 eV, and the 2LO-phonon replicas at 3.22–3.24 eV. The PL band at 3.359 eV in films comes also from the recombination of D^0^X excitons in ZnO crystallites, while the PL bands at 3.324 eV, 3.267 eV and 3.202 eV are related most likely to free-to-bound transitions due to some impurities in the ZnO crystallites.

Therefore, the annealing temperature of 400 °C is not enough for the production of single phase ZnMgO films by sol–gel spin coating. On the other hand, ZnMgO:ZnO composite films with ZnO nanoparticles embedded into the ZnMgO matrix are useful for fast electron transport and high charge balance in quantum dot light emitting diodes [22].

**Figure 6:**
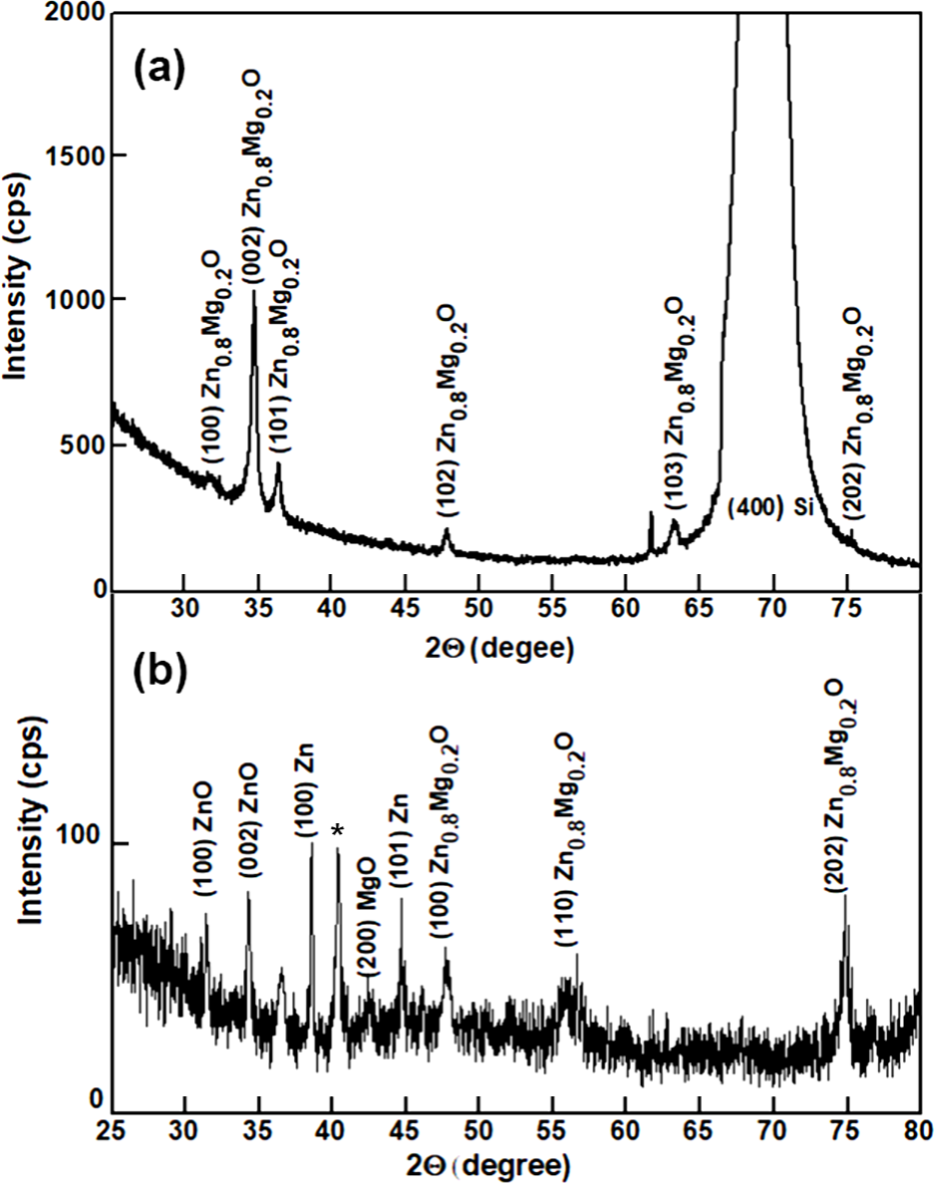
a) XRD pattern of a Zn_0.8_Mg_0.2_O film deposited by spin coating on a Si substrate and annealed at 500 °C. b) XRD pattern of a Zn_0.8_Mg_0.2_O film deposited by spin coating on a glass substrate and annealed at 400 °C.

The multiphase composition of films prepared by spin coating and annealed at temperatures lower that 450 °C was revealed by X-ray diffraction (XRD) analysis. As one can see from Figure 6b, reflexes related to ZnO inclusions (PDF Card No. 01-075-1533) are observed in the film annealed at 400 °C, along with those related to Zn_0.8_Mg_0.2_O (PDF Card No. 01-078-3032). The peak around 43° can be assigned to some trace of MgO, while those at 38.5° and 44° could be due to some Zn clusters [43]. A peak at 40.5° marked with an asterisk in Figure 6b was previously found in ZnO nanopowders prepared by the sol–gel method with zinc acetate dihydrate as a precursor [44]. In contrast to this, only peaks related to the Zn_0.8_Mg_0.2_O phase are observed in the film annealed at 500 °C. Silicon substrates were used for films annealed at temperatures higher than 500 °C to avoid softening of the glass substrate.

Table 3 summarizes the PL band position at 20 K and at room temperature in ZnMgO films prepared by sol–gel spin coating.

**Table 3:** The summarized PL band maximum in Zn_1−_*_x_*Mg*_x_*O films.

*x* value	PL band position at 20 K (eV)	PL band position at 300 K (eV)

0.00	3.36	3.30
0.05	3.45	3.39
0.10	3.52	3.45
0.15	3.53	3.50
0.25	3.56	3.50
0.40	3.56	3.53

**Figure 7:**
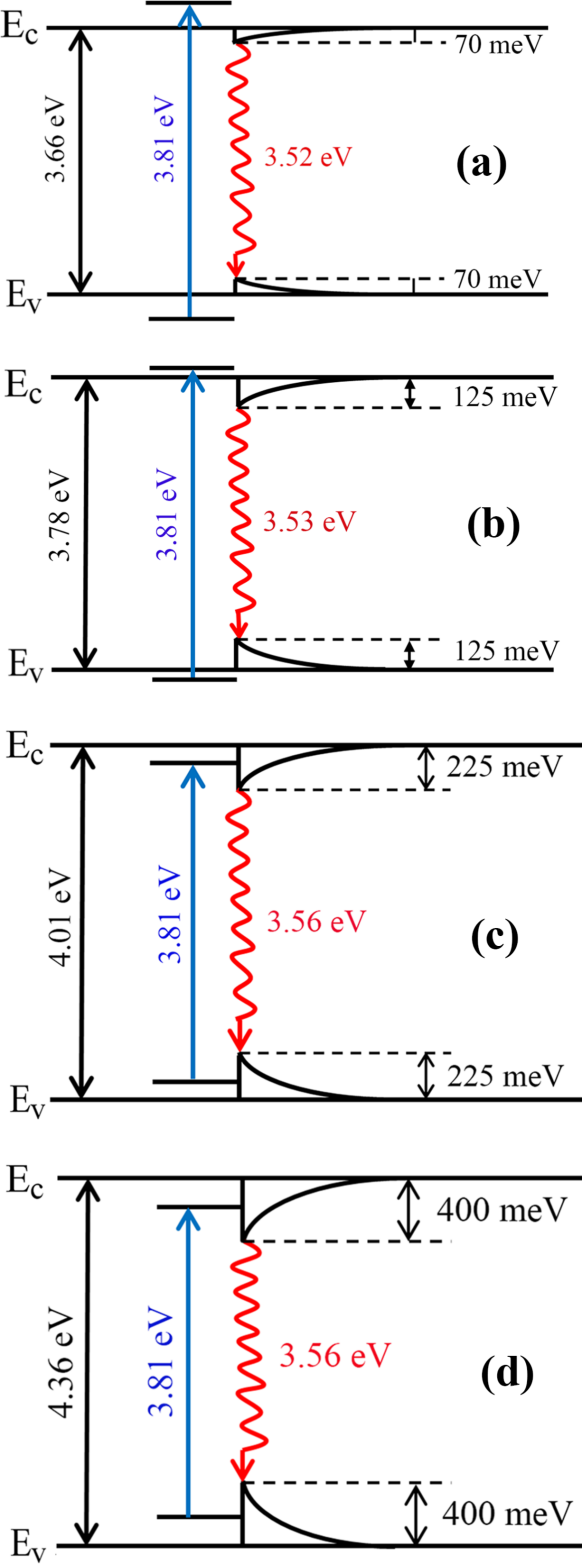
A model for the band tails distribution at 20 K in Zn_1−_*_x_*Mg*_x_*O films with the *x* value composition of a) 0.10; b) 0.15; c) 0.25 and d) 0.40.

A model for the band tail distribution and the PL position at 20 K is proposed in Figure 7 for ZnMgO films.

For the films of Zn_0.90_Mg_0.10_O and Zn_0.85_Mg_0.15_O, the laser line excitation energy is higher than the bandgap, while for the films of Zn_0.75_Mg_0.25_O and Zn_0.60_Mg_0.40_O the photon excitation energy is lower that the bandgap, and the luminescence is excited by transitions between the states from the band tails. After excitation, the carriers relax to the minimum possible energy in the band tails, which determines the spectral position of the PL band. With increasing *x* value from 0 to 0.40, the deepness of band tails in the gap increases to about 400 meV.

One can see from Figure 4 and Figure 5 that narrow emission lines related to resonance Raman scattering (RRS) are present in the emission spectrum from the ZnMgO films in addition to the broad PL bands, which is indicative of the high optical properties of the films produced by sol–gel spin coating. RRS from solids can be observed if the energy of the incoming or scattered photons matches real electronic states in the material. One refers to incoming and outgoing resonance, respectively [38,45–47]. Taking into account the band gap value and the width of band tails in ZnMgO thin films with various compositions at temperatures at which the emission spectra were measured (20 K and 300 K), and their correlations with the energy of the incident excitation photons (3.81 eV) and the energy of photons scattered by 1LO (3.74 eV) and 2LO (3.67 eV) phonons, Table 4 summarizes the conditions of RRS at the respective temperatures.

**Table 4:** The conditions of resonance Raman scattering in ZnMgO films for various compositions and temperatures.

Thin film composition	*T* = 20 K	*T* = 300 K

Zn_0.90_Mg_0.10_O	Bandgap (3.66 eV) in resonance with the 2LO scattered photon (3.67 eV). (Outgoing resonance)	No RRS lines
Zn_0.85_Mg_0.15_O	Bandgap (3.78 eV) in resonance with the incident photon (3.81 eV). (Ingoing resonance)	Bandgap (3.70 eV) in resonance with the 1LO scattered photon (3.74 eV). Band tails in resonance with the 2LO scattered photon (3.67 eV). (Outgoing resonance).
Zn_0.75_Mg_0.25_O	Band tails in resonance with the incident photon and with the 2LO scattered photon. (Combined resonance)	Band tails in resonance with the incident photon. (Ingoing resonance)
Zn_0.60_Mg_0.40_O	Band tails in resonance with the incident photon and with the 1LO scattered photon. (Combined resonance)	Band tails in resonance with the incident photon and with the 1LO scattered photon. (Combined resonance)

Therefore, according to Table 4, clear 2LO RRS peaks are observed in Figure 5b for the Zn_0.90_Mg_0.10_O and Zn_0.75_Mg_0.25_O samples at low temperature, and a 1LO RRS peak is found in Figure 5a for the Zn_0.75_Mg_0.25_O sample at room temperature. For the sample Zn_0.85_Mg_0.15_O, the 2LO RRS peak is observed at low temperature in Figure 4b, and the peaks corresponding to 1LO RRS and 2LO RRS lines are revealed in Figure 4a at room temperature. Finally, lines corresponding to 1LO RRS and 2LO RRS processes are observed in Figure 4a and Figure 4b due to the interaction of the large band tails with both the incident and scattered photons in the Zn_0.60_Mg_0.40_O sample.

The prepared ZnMgO thin films were tested for photodetector applications in the metal–semiconductor–metal (MSM) design configuration with coplanar metal Pd contacts in our previous paper [29]. The films demonstrated photosensitivity under UV light irradiation, where the photosensitivity was much higher in samples prepared by spin coating as compared to those prepared by aerosol spray pyrolysis. Additionally, the resistivity of films deposited by spin coating was found to be much higher. Apart from that, a long duration relaxation of photoconductivity was shown to be characteristic for films prepared by spin coating, while a fast response to irradiation was observed in samples prepared by aerosol spray pyrolysis.

Similar to the issues about the influence of the technology on the morphology of films discussed above, we suppose that the difference in the electrical parameters of films prepared by the two methods is determined by the specific features of the technology. The concentration of unintentionally introduced impurities and intrinsic defects is different with the two methods, particularly due to different numbers of technological steps and the different temperature of the substrate during the deposition processes. In our opinion, the higher resistivity of the films prepared by spin coating as compared to those obtained by spray pyrolysis indicates a higher degree of conductivity compensation, due to the higher concentration of acceptor levels introduced during spin coating.

**Figure 8:**
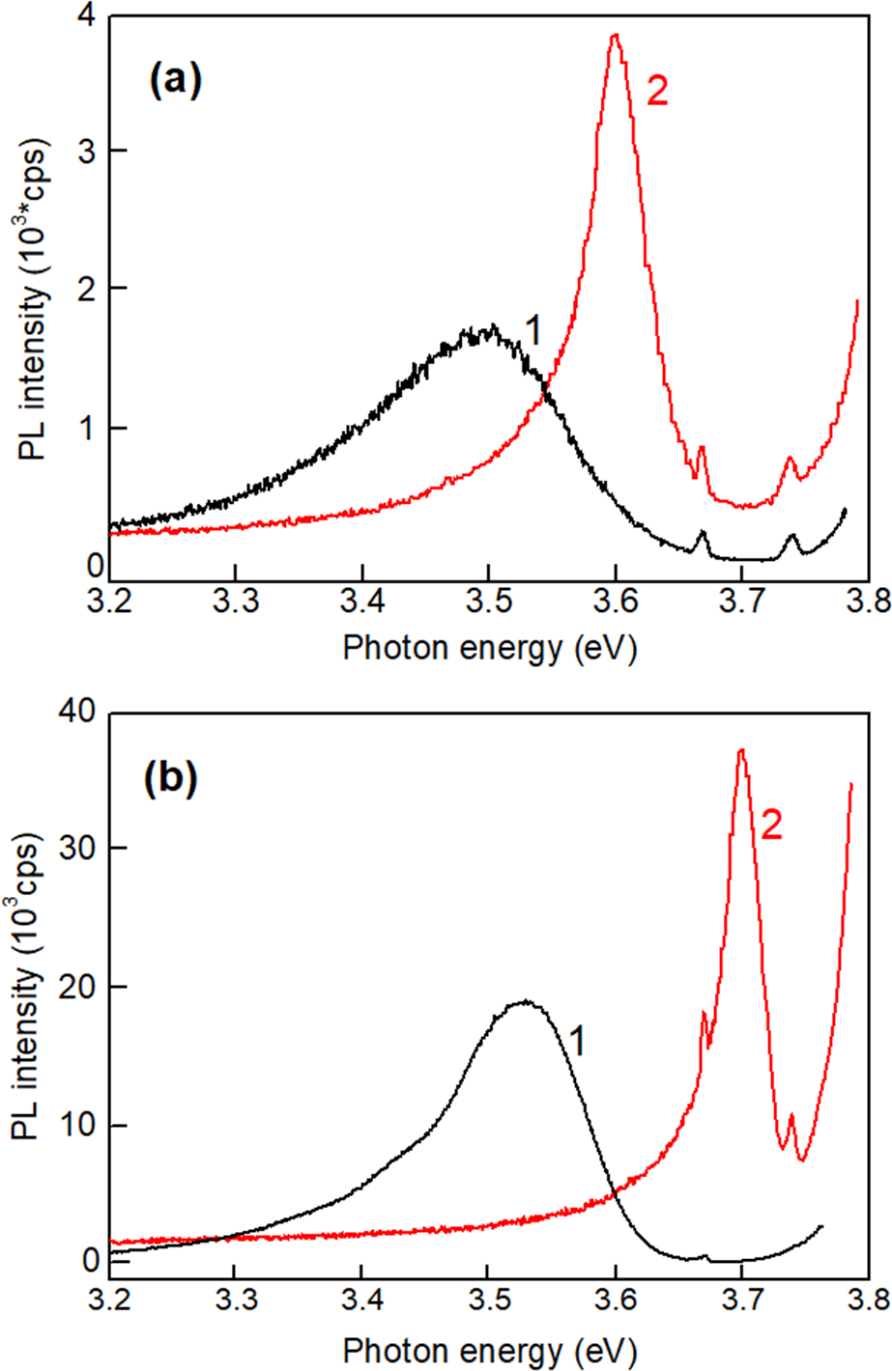
PL spectra of Zn_0.85_Mg_0.15_O films by spin coating (curve 1) and aerosol spray pyrolysis (curve 2) measured a) at room temperature and b) at 20 K with a linear intensity axis.

Long duration relaxation of photoconductivity and persistent photoconductivity was previously observed in highly doped and compensated semiconductors [39], porous semiconductors [48] and solid solutions [40]. The origin of these phenomena was assumed to be the same in different materials, and it was attributed to random local-potential fluctuations. As mentioned above, the random local-potential fluctuations are also responsible for the emergence of broad PL bands in the near bandgap spectral region. These potential fluctuations lead to the formation of potential barriers, which have to be overcome for the recombination of photoexcited carrier to occur during the relaxation processes. On the other hand, the mechanisms for attaining such potential fluctuations were found to be different. In highly doped semiconductors, the amplitude of potential fluctuations is determined by the degree of doping and conductivity compensation. In porous semiconductors the amplitude is determined by the degree of porosity, while it is a function of local fluctuations of the composition in solid solutions, including ZnMgO. The observation of the long duration component in the relaxation of photoconductivity in ZnMgO films deposited by spin-coating corroborates the data deduced from the analysis of photoluminescence spectra. At the same time, the lack of such a component in films prepared by aerosol deposition may be interpreted as reduced local composition fluctuations and lower local-potential fluctuations in such films. This statement is corroborated by the comparison of PL spectra of films prepared by the two methods. For example, Figure 8 compares the PL spectra of two films with the composition of Zn_0.85_Mg_0.15_O prepared by spin coating and aerosol spray pyrolysis, measured at low temperature (20 K) and room temperature.

One can see that the PL band in the film prepared by spay pyrolysis is much narrower as compared to the band in the film prepared by spin coating, and it is shifted to higher photon energy, i.e., closer to the bandgap position. Apart from that, the luminescence in films prepared by aerosol spray pyrolysis is not excited, when the *x* value is higher than 0.15. This observation indicates that the band tails are very narrow, if there are any. It could also be that the local-potential fluctuations in films prepared by spray pyrolysis are due to inhomogeneous distribution of intrinsic defects or unintentional doping impurities, as previously observed in undoped [38,49] or Cu, Ni, Co, or Al doped [46,50] ZnO materials, instead of local composition fluctuations.

Usually, the full width at half maximum (FWHM) of the PL band in ZnO with carrier concentration in the range of 10^18^ cm^−3^ to 10^19^ cm^−3^ is less than 50 meV. The concentration should be in the range of 10^20^ cm^−3^ to 10^21^ cm^−3^ to reach a FWHM value of 200 meV, i.e., the material should be highly conductive. On the other hand, the FWHM of PL bands in films prepared by spin coating reaches values of 200 meV, while the material is highly resistive, as mentioned above. This means that the formation of large band tails in films prepared by spin coating cannot be attributed to doping with impurities or to intrinsic defects, but to local composition fluctuations. The observed PL band also cannot be attributed to low concentration impurities or intrinsic defects since the PL bands related to such impurities in the region of the absorption edge are narrow (they are due to either free-to-bound transitions or donor-acceptor transitions) [41]. Wider PL bands related to impurities are observed in the visible spectral range, but this is not the subject of this paper.

**Figure 9:**
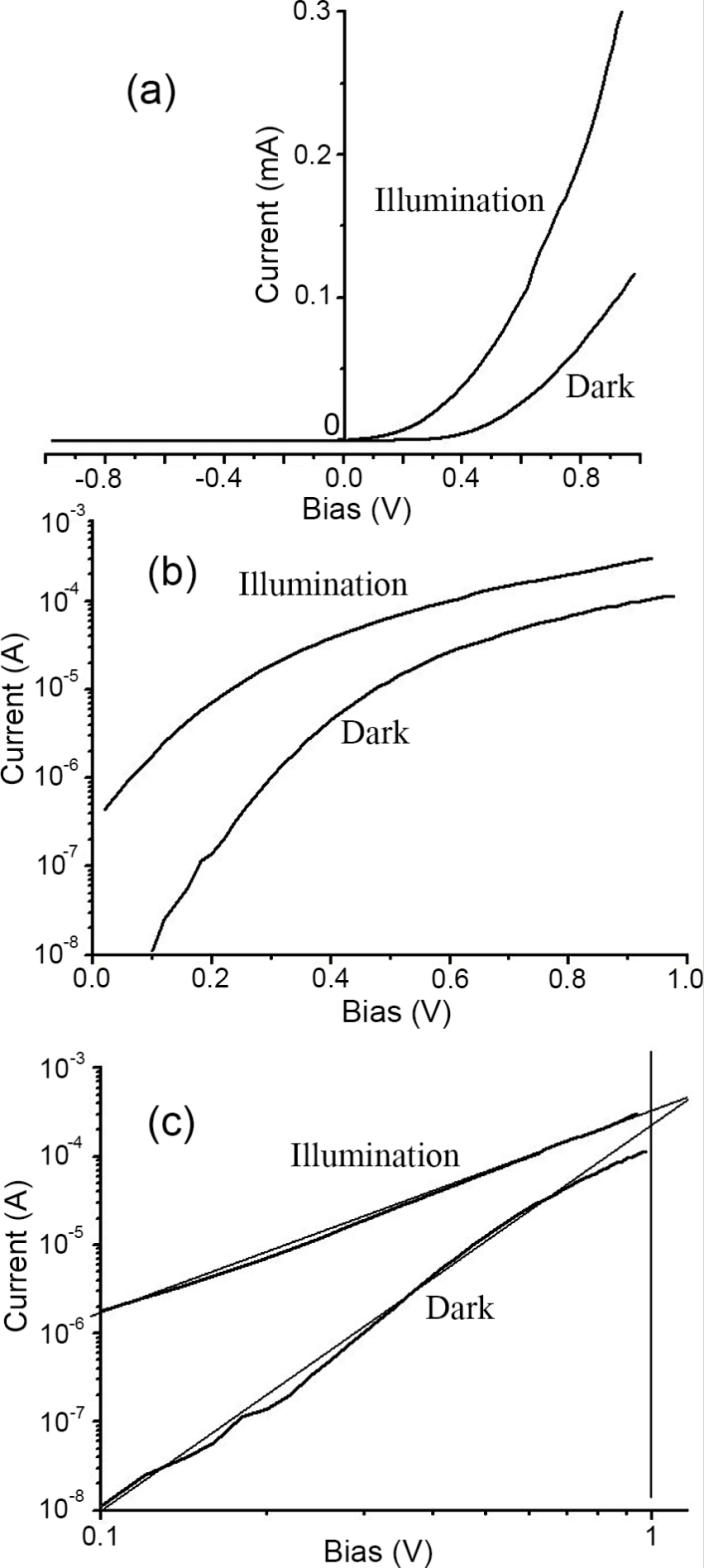
Current–voltage characteristics in dark and under UV illumination for a p-Si/n*-*Zn_0.9_Mg_0.1_O heterostructure plotted on linear (a), semi-logarithmic (b) and double logarithmic coordinates (c).

**Figure 10:**
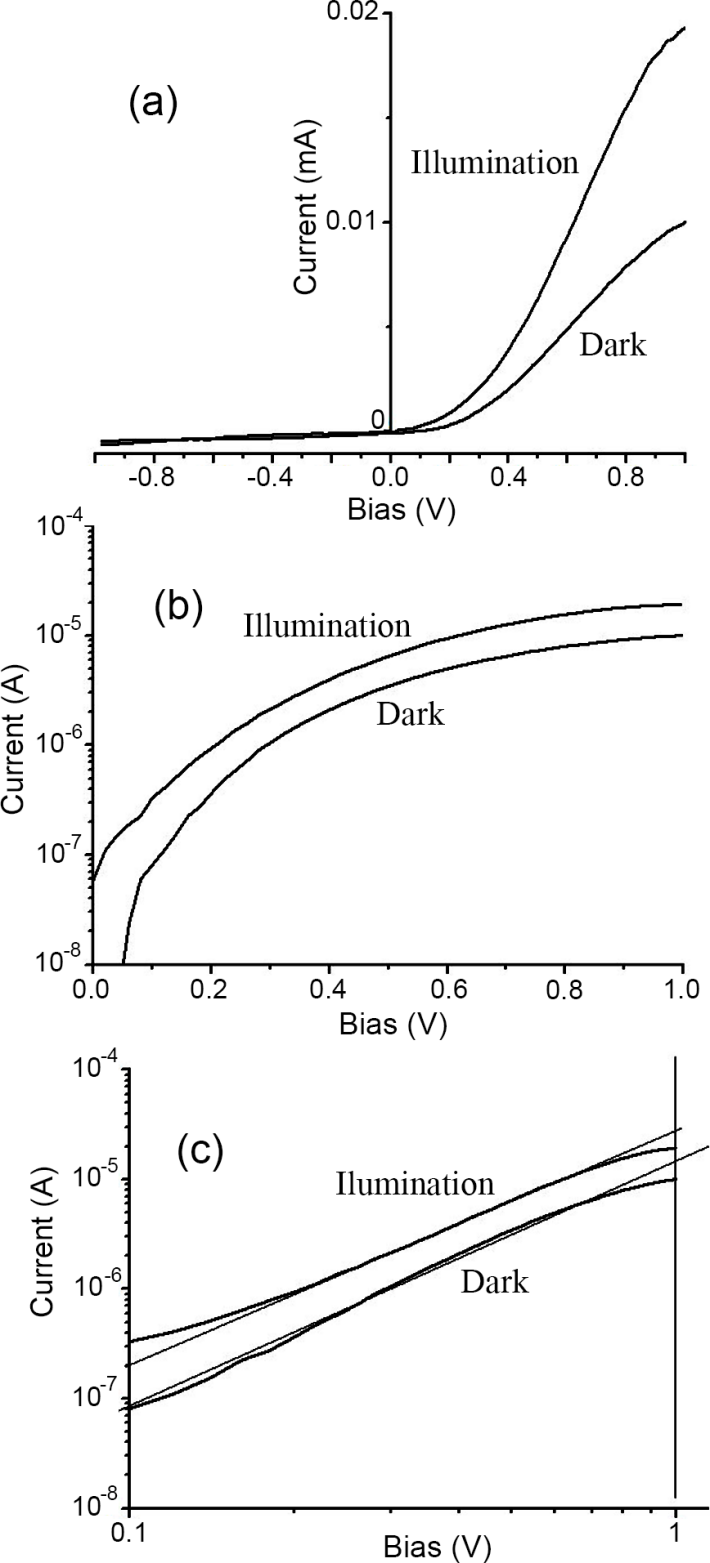
Current–voltage characteristics in dark and under UV illumination for a p-Si/n-Zn_0.6_Mg_0.4_O heterostructure plotted on linear (a), semi-logarithmic (b) and double logarithmic coordinates (c).

Finally, the ZnMgO films deposited on p-type Si substrates were tested for photodetector applications in a heterostructure design with a metallic contact deposited on the n-ZnMgO film and another contact on the p-type Si substrate. Figure 9 and Figure 10 compare the current–voltage characteristics of p-Si/n-Zn_1−_*_x_*Mg*_x_*O heterojunctions for two films deposited by spin coating with *x* values of 0.10 (Figure 9) and 0.40 (Figure 10). One can see from Figure 9b and Figure 10b that in both cases the current–voltage characteristic does not fit the classical formula for a p–n junction, 
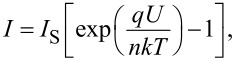
 which should be a straight line for the forward bias in the semi-logarithmic coordinates. On the contrary, the characteristics are fit to straight lines in the double logarithmic coordinates (Figure 9c and Figure 10c). Moreover, the investigated heterojunctions work as photodetectors at forward bias, while a classical p–n junction should function as a photodetector at reverse bias.

Since the current–voltage characteristics are fit to straight lines in the log–log coordinates, it means that they correspond to a power function *I* ∝ *U*^n^, according to the Lampert theory [51]. For the heterojunction with a Zn_0.9_Mg_0.1_O film in the dark, the *n* value is about 4, while it becomes equal to 2 under illumination, which corresponds to the space charge limited (SCL) current injection according to the Mott–Gurney (MG) law [52]. For the heterojunction with a Zn_0.6_Mg_0.4_O film, the current–voltage characteristics fit to the MG law both in the dark and under illumination (see Figure 10c). These observations suggest that the investigated heterojunctions work at forward bias as injection photodiodes [53–54].

A more detailed investigation of photodetectors developed on ZnMgO films, including correlations between PL and sensing properties, is in progress in our laboratory, but the results will be published in a separate paper.

## Conclusion

The results of this study demonstrate the preparation of ZnMgO thin films by spin-coating on Si substrates with homogeneous morphology at the macrosopic level. However, compositional fluctuations of the alloy are deduced at the microscopic level from the investigation of photoluminescence spectra. The local potential fluctuations induced by compositional fluctuations lead to the formation of deep band tails in the gap, which make it possible to excite photoluminescence with under-bandgap photon energies. The potential fluctuations also result in a long duration relaxation of photoconductivity in photodetectors prepared on these films. The p-Si/n-Zn_1−_*_x_*Mg*_x_*O heterojunction photodetectors work at forward bias as injection photodiodes. The performed investigations demonstrate that post-deposition annealing at 500 °C is needed for the production of wurtzite single crystallographic phase Zn_1−_*_x_*Mg*_x_*O films in the composition range of *x* = 0.00–0.40. Annealing at higher temperature leads to morphology degradation, while thermal treatment at lower temperatures is not enough for producing single phase films, ZnO nanoparticles being embedded into the ZnMgO matrix, as deduced from photoluminescence spectra and XRD analysis. Nevertheless, such films could also find specific applications, for instance in quantum dot light emitting diodes.

## Experimental

ZnMgO thin films were prepared by spin coating from sol–gel solutions containing Zn(CH_3_CO_2_)_2_ and Mg(C_2_H_3_O_2_)_2_ acetates in respective proportions dissolved in 20 mL of 2-methoxyethanol + 0.5 mL of diethanolamine (DEA). 0.35 M solutions with Mg/Zn from 0 to 2/3 were prepared in an ultrasonic bath for 30 min at a temperature of 50–60 °C. Spin coating was performed at room temperature on glass or (100) on p-Si substrates in multiple coating cycles at a rotational speed of 2000 rpm with the rotation taking 20 s followed by drying the coated layer at 150 °C for 10 min. After the deposition of 10 layers, the sample was treated at a temperature in the range of 400–650 °C in air for one hour. For the purpose of comparison, thin films were also prepared by the aerosol spray pyrolysis method. A solution of zinc acetate dihydrate with 99.999% purity and magnesium acetate tetrahydrate with purity ≥ 99%, both purchased from Sigma-Aldrich, dissolved in ethanol (C_2_H_5_OH), was sprayed onto the substrate using a homemade sprayer with an O_2_ gas flow. The substrate was heated in the temperature range of 400 °C to 650 °C during the deposition. 0.35 M zinc acetate and magnesium acetate solutions were mixed in an ultrasonic bath in various proportions to produce ZnMgO films with Mg content from 5% to 40%. A distance of 18 cm was experimentally chosen between the sprayer and the heated substrate in view of producing a uniform coverage of the film on the substrate. The solution was injected into the oxygen gas flow by means of a syringe controlled by a stepper motor (Jova Solutions TIMS-0201™), operated by a computer. The produced film thickness is determined by the rate of precursor solution injection and the duration of deposition process. Usually, an injection rate of 0.33 mL/min was used, and the deposition process lasted for 15 min.

The morphology and chemical composition microanalysis of the produced films were studied using a Zeiss Sigma SEM, Hitachi SU 8230, equipped with tools for energy dispersive X-ray analysis (EDAX). Atomic force microscopy (AFM) measurements were performed in tapping mode with a SOLVER Next (NT-MDT) instrument equipped with cone-shaped tips from monocrystalline silicon (tip radius ≈ 10 nm) on cantilevers with a stiffness of about 17 N/m. The root mean square (RMS) roughness parameters were calculated from the acquired topographic images using image processing software.

The continuous wave (cw) PL was excited by the 325 nm line of a He–Cd Kimmon laser and analyzed with a double spectrometer, ensuring a spectral resolution better than 1 meV. The samples were mounted on the cold station of a LTS-22-C-330 optical cryogenic system. The current–voltage characteristics and the photocurrent of the photodetector structures were measured with a Keithley 2400 Source Meter Unit (SMU).
